# Association of inflammation cytokines with cognitive function in first-episode major depressive disorder

**DOI:** 10.3389/fpsyt.2024.1473418

**Published:** 2025-01-22

**Authors:** Yan Qing Xi, Zong Qi Wang, Guo Juan Li, Zhuo Qun Hao, Jia Hui Nie, Jin Xiang Li, Yu Ting Tan, Xiao Dong Hu, Gen Wei Wang, Sha Liu, Yan Fang Wang

**Affiliations:** ^1^ Department of Psychiatry, First Hospital/First Clinical Medical College of Shanxi Medical University, Taiyuan, China; ^2^ Shanxi Key Laboratory of Artificial Intelligence Assisted Diagnosis and Treatment for Mental Disorder, First Hospital of Shanxi Medical University, Taiyuan, China; ^3^ School of Public Health, Shanxi Medical University, Taiyuan, China; ^4^ First Clinical Medical College, Shanxi Medical University, Taiyuan, China; ^5^ School of Humanities and Social Sciences, Shanxi Medical University, Taiyaun, China; ^6^ Department of Microbiology and Immunology, School of Basic Medical Sciences, Shanxi Medical University, Taiyuan, China

**Keywords:** antidepressant, attention, immunology, major depressive disorder, memory

## Abstract

**Objective:**

Abnormal cognitive functioning is a core symptom of Major Depressive Disorder (MDD) and is strongly correlated with MDD prognosis. Current studies suggest that the occurrence of MDD may be related to oxidative stress-induced inflammation, hypothalamic-pituitary-adrenal axis disorders, diminished monoamine function and microbe-brain-gut axis, among other pathways. In recent years, the relationship between the immune-inflammatory response and MDD has been a hot topic of research, but how the relationship between immunoinflammation and cognitive function is manifested in MDD is still unclear. In this study, we examined cognitive function characteristics, serum inflammatory factors, brain-derived neurotrophic factor, and their correlations before and after pharmacological treatment(paroxetine hydrochloride tablets) in patients with first-episode major depressive disorder, aiming to identify objective biomarkers for cognitive function assessment.

**Methods:**

We included 22 patients with first-episode major depressive disorder and 27 healthy volunteers from the community during the same period. The Hamilton Depression Scale-17 (HAMD-17) assessed the severity of depressive symptoms at baseline and after 8 weeks of treatment. The Repeatable Battery for the Assessment of Neuropsychological Status(RBANS) evaluated cognitive function, and serum samples were collected to determine levels of inflammatory and neurotrophic factors at these two time points. For healthy volunteers, only HAMD-17 scale scores, RBANS scale scores, and serum samples were taken at baseline. Spearman’s correlation analyzed the relationship between inflammatory factors, neurotrophic factors, and cognitive function. Multiple linear regression determined factors affecting cognitive function in first-time patients.

**Results:**

Baseline findings indicated that patients’ IL-6 and TNF-α levels exceeded those of healthy individuals, while their IFN-α levels were below; their scores in language, attention, delayed memory, and the RBANS scale were also lower than healthy counterparts. Post-treatment, patients’ BDNF, IL-6, and TNF-α levels remained higher than those of healthy subjects, and their IFN-α levels were still lower; their language and attention scores were also inferior. Association analyses revealed an association between BDNF and visuospatial/constructional ability scores and language scores in patients with MDD at baseline, and a positive relationship between TNF-α and attention score. Multiple regression analysis indicated an association between TNF-α levels and attention scores in MDD patients at baseline.

**Conclusions:**

Our study concludes that TNF-α and BDNF correlate with cognitive function in MDD at baseline, and furthermore, TNF-α could potentially serve as an objective biomarker to support the assessment of attentional function at baseline.

## 1 Introduction

Major Depressive Disorder (MDD) is a common, highly disabling psychiatric disorder severely limiting psychosocial functioning and decreasing the quality of life (1, 2). MDD is characterized by a depressed mood and diminished interest as core symptoms, it is often accompanied by cognitive impairment and somatic physical symptoms. Cognitive impairment is characterized by attention deficits, reduced verbal and non-verbal learning ability, impairment of short-term and working memory, as well as impaired visual and auditory processing speed (3), and is strongly correlated with the prognosis of MDD (4). Most patients with MDD experience cognitive impairment, and some struggle to recover from these cognitive deficits even after the mood symptoms have abated (5, 6). Cognitive impairment is less severe in MDD than in schizophrenia and bipolar disorder, making clinical assessment more challenging. Currently, the evaluation of cognitive function in MDD patients is mainly through the assessment of scales, and the search for objective biomarkers that can reflect the status of cognitive function can help to identify the cognitive impairment of MDD patients as well as to assess the recovery of cognitive function after treatment.

The mechanisms underlying cognitive impairment in MDD patients are intricate. Current studies suggest that increased oxidative stress-induced inflammation (7), hypothalamic-pituitary-adrenal axis disorders (8, 9), diminished monoamine function (10) and microbe-brain-gut axis (11), among other factors, may contribute to cognitive impairment. In recent years, the mechanism of immunoinflammation has become a hot topic, but how the relationship between immunoinflammation and cognitive function is manifested in MDD is still unclear. The immune-inflammatory response is mainly mediated by cytokines (especially inflammatory cytokines).During acute inflammation, inflammatory agents infiltrate the central nervous system via the blood-brain barrier. This results in various abnormal behavioral, cognitive, and emotional responses in MDD patients (12). Thus, inflammatory agents might serve as predictors of treatment response in MDD (13). Inflammatory factors mainly include the interleukin family, interferon, tumor necrosis factor, and so on.IL-6, produced by immune cells, plays a role in modulating immune responses and other functions. TNF-α is a versatile signaling molecule vital to both immunity and the standard operations of the central nervous system. McAfoose et al. (14) proposed that inflammation-driven cognitive processes, triggered by factors like IL-1,IL-6, and TNF-α,are notable in both healthy individuals and those with long-term neuropsychiatric disorders, such as major depression and dementia. This makes these inflammatory factors promising targets for therapeutic interventions. Elevated IL-6 levels are present in those with mild cognitive impairment and Alzheimer’s disease, potentially heightening the risk of cognitive decline (15). Furthermore, in addition to the interleukin family and tumor necrosis factor, rats in an IFN-α induced depression model displayed heightened central and peripheral inflammatory markers, diminished brain-derived neurotrophic factor, and compromised learning capability (16, 17).

Moreover, brain-derived neurotrophic factor (BDNF) is widely present in the central nervous system and can promote neuronal cell survival, enhancing synaptic plasticity and neurogenesis. Their signaling pathways predominantly involve the phosphoinositide 3-kinase (PI3K)-AKT (also known as protein kinase B, PKB) pathway and the mitogen-activated protein kinase (MAPK) extracellular signal-regulated kinase (ERK) pathway (18). These pathways mediate neuronal survival, proliferation, and differentiation, vital for maintaining normal cognition and learning functions (19). Evidence suggests that BDNF levels in MDD patients are lower than in healthy individuals (20), and BDNF may influence the restoration of cognitive function in those with MDD (21).

Given the intricate clinical features and co-morbidities of MDD (22), these variables introduce variability in cognitive assessments for MDD patients (23). Thus, the selection of stable and reliable biological markers to assist in cognitive function evaluation and to guide individualized treatment efficacy (24) is a pressing issue.

In this study, we began by examining inflammatory factors IL-6, TNF-α, IFN-α, and BDNF. We explored the traits of cognitive function, inflammatory agents, and brain-derived neurotrophic factors, and their associations before and after medication treatment in patients with first-episode major depressive disorder. Our aim was to identify biomarkers that could assist in evaluating MDD patients’ cognitive function and predicting improvements in cognition, providing a foundation for clinical treatment.

## 2 Materials and methods

### 2.1 Participants

Twenty-two patients with first-episode major depressive disorder who attended the First Hospital of Shanxi Medical University from June 2022 to December 2022 were selected as study subjects, along with 27 healthy volunteers recruited from the community during the same period. The study subjects were all Asian.

Inclusion criteria were as follows: 1) Meeting the Diagnostic and Statistical Manual of Mental Disorders, 5th Edition (DSM-5) (25) “Major depressive disorder (current episode)” diagnostic criteria, not currently receiving treatment; 2) Age 18-55 years, han ethnic group; 3) HAMD-17 score ≥ 17(indicates that subjects may be mildly or moderately depressed); 4) Right-handedness. Inclusion criteria for healthy volunteers: 1) Age 18-55 years, han ethnic group; 2) Not meeting the diagnostic criteria of any DSM-5 psychiatric disorders; 3) HAMD-17 score <7 (indicates that the subject does not have depressive symptoms); 4) No family history of psychiatric disorders in two or three generations. Exclusion criteria for all subjects: 1) Those with comorbid organic mental disorders; 2) Exclusion of patients with serious diseases of the cardiovascular system, liver, kidneys, and other organs, i.e., not associated with somatic diseases; 3) Patients with bipolar disorder or suicidal ideation; 4) Pregnant or lactating women.

All study subjects voluntarily cooperated with this investigation and signed the informed consent form, and the study was reviewed and approved by the Ethics Committee of the First Hospital of Shanxi Medical University (Approval No.K055).

### 2.2 Research design

This study was an 8-week trial with assessment visits at baseline (0 weeks,0 w) and at the end of the trial (8 weeks,8 w). Baseline serum samples were collected from MDD patients and healthy subjects to assess the general information. The clinical case observation report form for affective disorders, including gender, age, and years of education, etc., which was uniformly developed by the Department of Psychiatry of the First Hospital of Shanxi Medical University, HAMD-17 and RBANS scales were used for evaluation. Selective Serotonin Reuptake Inhibitors (SSRI) are commonly prescribed antidepressants proven effective in alleviating depressive symptoms (26, 27). SSRIs mainly include fluoxetine, paroxetine, sertraline, fluvoxamine, citalopram, and escitalopram, while paroxetine inhibits 5-HT reuptake strongly (28, 29), has a fast onset of action, and rapidly improves depression and sleep, so paroxetine hydrochloride tablets were chosen as the therapeutic drug in this study. Patients were treated with paroxetine hydrochloride tablets for 8 weeks after enrollment. The initial dose was 10 mg/d, increased to 20 mg/d in week 1. After 1-2 weeks of administration, the dose was increased by 10-20 mg depending on the patient’s condition, up to a maximum of 40 mg/d. At the end of 8 weeks of treatment, serum samples were collected from MDD patients and assessed again with HAMD-17 and RBANS scales. Healthy controls did not receive any treatment and only participated in baseline data collection.

### 2.3 Collection and testing of blood samples

From MDD patients, 5-6 ml of fasting peripheral venous blood was collected at 0w and 8w.In contrast, we only collected the fasting peripheral venous blood of HC at 0w. This was left to stand for 2 hours at room temperature before being centrifuged at 4℃, 3500 r/min for 10 mins. The supernatant after centrifugation was transferred into EP tubes and stored at -80°C. The BDNF and serum inflammatory cytokines levels were measured using enzyme-linked immunosorbent assay kit for IL-6, TNF-α, IFN-α, and BDNF. The procedure was carried out strictly according to the reagent instructions. The assay utilized the Burton ELx808 enzyme labeling instrument from the United States, with the thermostat being MIR-262 from Sanyo. The kit was supplied by CLOUD-CLONE CORP.WUHAN. The coefficient of variation in intra-assay: BNDF,IL-6,IFN-α,TNF-α<10%. The coefficient of variation in inter-assay: BNDF,IL-6,IFN-α,TNF-α<12%.

### 2.4 Scale

#### 2.4.1 Hamilton Depression Scale-17 (HAMD-17)

Compiled by Hamilton in 1960, HAMD-17 (30) is a frequently utilized scale in Chinese clinics to assess the severity of depression. This self-rating scale encompasses five factors: anxiety/somatic, weight, cognitive impairment, blockage, and sleep disorder, with a total of 17 items. Among them (1), anxiety/somatization consists of 6 items, including anxiety-psychic, anxiety-somatic, somatic symptoms-gastrointestinal, somatic symptoms-general, hypochondriasis and insight; (2) weight, one item of weight loss; (3) cognitive impairment consists of 3 items, including feelings of guilt, suicide, and agitation; (4) blockage consists of 4 items, including depressive mood, work and interest, retardation, and genital symptoms; and (5) sleep disorder consists of 3 items, including insomnia-initial, insomnia-middle, insomnia-delayed. Most items in the HAMD-17 scale were rated on a 5-point scale from 0-4. Each scale is rated as (0) none; (1) mild; (2) moderate; (3) severe; and (4) very severe. A few items were scored on a 3-point scale from 0-2. Each scale is rated as (0) none; (1) mild to moderate; and (2) severe. A total score of <7 on the HAMD-17 scale indicates that no depressive symptoms are present; a total score of ≥17 on the HAMD-17 scale may be mild or moderate depression; and a total score of ≥24 on the HAMD-17 scale may be severe depression. A higher HAMD-17 score indicates a more severe degree of depression.

#### 2.4.2 The Repeatable Battery for the Assessment of Neuropsychological Status (RBANS)

The RBANS (31) used in Chinese clinical settings, the RBANS evaluates cognitive function. It consists of 12 items divided into five subscales: immediate memory, visuospatial/constructional, language, attention, and delayed memory. Among them, ① Immediate memory: including vocabulary memorization (scoring range:0-40 points) and story memorization (scoring range:0-24 points) 2 tasks; ② visuospatial/constructional: including graphics copying (scoring range:0-20 points) and line localization (scoring range:0-20 points) 2 tasks; ③ language: including picture naming (scoring range:0-10 points) and fluent speech (scoring range:0-40 points) 2 tasks; ④ attention: including digital breadth (scoring range:0-16 points) and encoding (scoring range:0-89 points) 2 tasks; ⑤ delayed memory: including vocabulary recollection(scoring range:0-10 points),vocabulary recognition(scoring range:0-20 points),story recollection (scoring range:0-12 points) and graphics recollection (scoring range:0-20 points) 4 tasks. The raw scores of these subscales are standardized based on age using the conversion table in the RBANS Stimulation Manual. These standardized scores are then summed and converted into a consolidated RBANS total score, with lower scores indicating poorer cognitive function.

### 2.5 Statistical analysis

Data analysis was performed using SPSS 25.0. Measures fitting a normal distribution were represented as mean ± standard deviation ( $\underset{-}{x}$ *± s*). Comparisons between groups were conducted using the independent samples t-test or one-way ANOVA. Measures with a skewed distribution were presented as Median (IQR 25-75), with group comparisons made using the Mann-Whitney U test. Comparisons between the baseline MDD and HC groups and between the post-treatment MDD and HC groups were performed using the Kruskal-Wallis H test. Baseline MDD and post-treatment MDD comparisons using the Friedman test. Two-way group comparisons were adjusted using the Bonferroni method. Count data were presented as relative numbers, with group comparisons done using the *χ^2^* test. The Spearman rank correlation assessed the relationship between inflammatory factors, neurotrophic factors, and cognitive functions. Multiple linear regression evaluated the cognitive function influencers with the RBANS scale score and individual factor scores as dependent variables, and BDNF, IL-6, TNF-α, and IFN-α as independent variables. A two-sided *P*<0.05 was deemed statistically significant.

## 3 Results

### 3.1 Demographic and clinical data

Comparison of age, gender, years of education, and marital status between the MDD group and the HC group revealed no statistically significant difference (*p*>0.05). However, for all other factors except weight, there were significant differences (*p*<0.05) between the MDD and HC groups in terms of anxiety/somatic factor, cognitive impairment factor, clogging factor, sleep disorders factor, and total HAMD score. The scores for all these factors in the MDD group were higher than those in the HC group (**Table 1**).

**Table 1:** Demographic characteristics of MDD and HC.

Demographic characteristics	MDD (*n*=22)	HC (*n*=27)	*χ* ^2^/*t/u*	*P*
Age,Median (IQR27-75)	27 (23.75, 35)	31 (26, 33)	-1.471	0.141
Gender				
Male (%)	11 (50.0)	14 (51.9)	0.017	0.897
Female (%)	11 (50.0)	13 (48.1)		
Years of education,mean (SEM) years	13.36 (2.74)	14.96 (2.30)	3.142	0.083
Marital Status				
Unmarried (%)	11 (50.0)	11 (40.7)	0.420	0.517
Married (%)	11 (50.0)	16 (59.3)		
HAMD-17				
Anxiety/Somatic,Median (IQR27-75)	7 (5, 8)	0 (0, 1)	-6.129	<0.001*
Weight,Median (IQR27-75)	0 (0, 1)	0 (0, 0)	-1.931	0.053
Cognitive impairment,Median (IQR27-75)	3 (3, 4)	0 (0, 0)	-6.096	<0.001*
Clogging,Median (IQR27-75)	6 (5, 7)	0 (0, 1)	-6.211	<0.001*
Sleep disorders,Median (IQR27-75)	3.5 (2.75, 5)	0 (0, 1)	-5.306	<0.001*
Total HAMD score,Median (IQR27-75)	19.5 (17, 22)	1 (0, 3)	-6.011	<0.001*

HAMD-17, 17-item Hamilton depression scale.

*p<0.05. Bolded text indicates that the value is statistically different.

### 3.2 Comparison of baseline inflammatory cytokines and BDNF between MDD and HC groups

**Figure 1:**
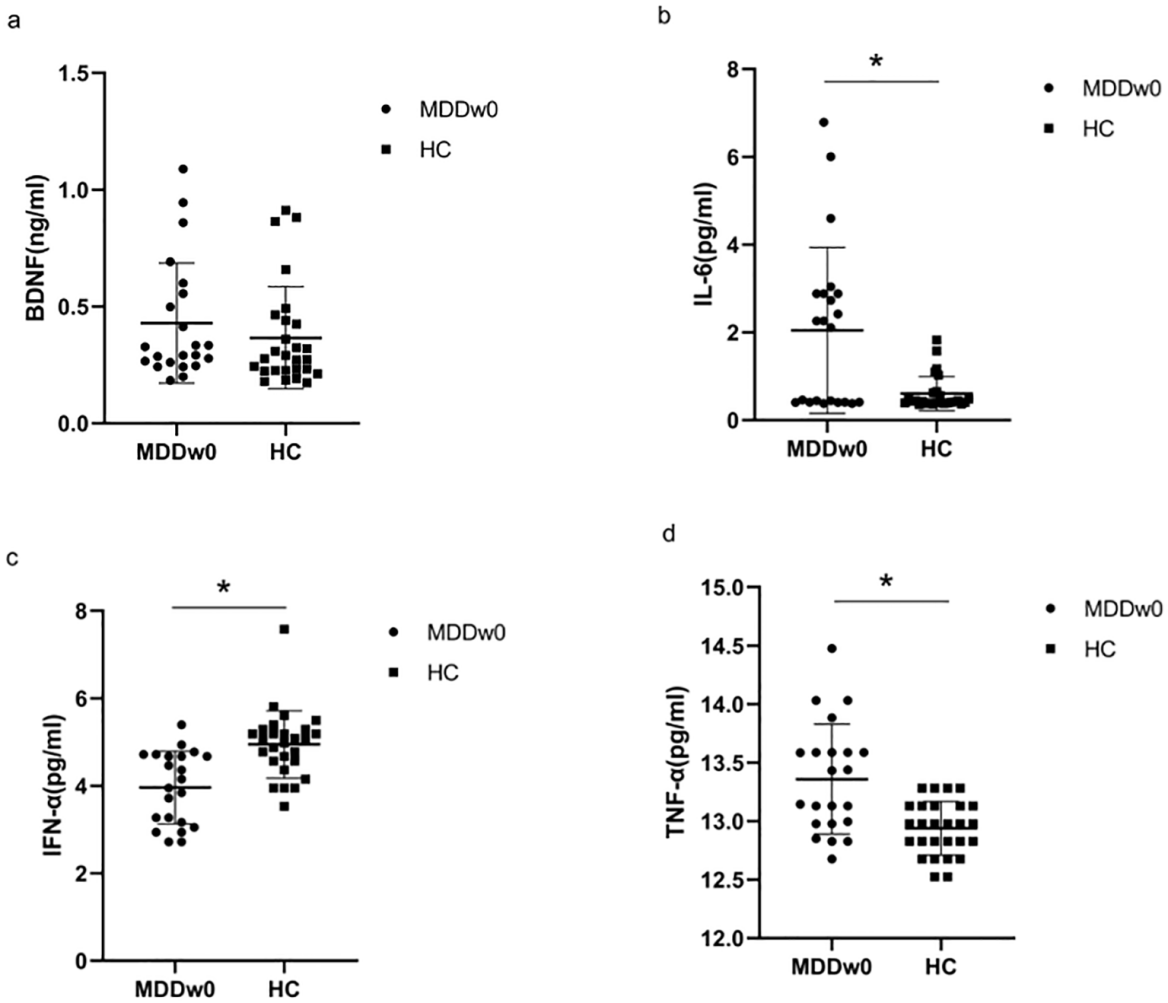
Comparison of baseline inflammatory cytokines and BDNF between MDD and HC groups. *p<0.05. **(A)** Comparison of baseline BDNF between MDD and HC groups. **(B)** Comparison of baseline IL-6 between MDD and HC groups. **(C)** .Comparison of baseline IFN-α between MDD and HC groups. **(D)** Comparison of baseline TFN-α between MDD and HC groups.

There was no significant difference in the BDNF levels between the MDD and HC groups (*p*>0.05). The levels of IL-6, TNF-α, and IFN-α between the two groups were statistically significant (*p*<0.05), with IL-6 and TNF-α levels in the MDD group higher than in the HC group, and IFN-α levels in the MDD group lower than in the HC group (**Table 2**, **Figure 1**).

**Table 2:** Comparison of baseline inflammatory cytokines and BDNF between MDD group and HC group.

Index	MDD (*n*=22) Median (IQR27-75)	HC (*n*=27) Median (IQR27-75)	*u*	*P*
BDNF(ng/mL)	0.31 (0.26, 0.57)	0.28 (0.23, 0.44)	-1.266	0.205
IL-6(pg/mL)	2.18 (0.41, 2.88)	0.43 (0.39, 0.62)	-2.646	0.008*
TNF-α(pg/mL)	13.29 (12.98, 13.59)	12.98 (12.83, 13.13)	-3.372	0.001*
IFN-α(pg/mL)	4.05 (3.13, 4.68)	5.08 (4.57, 5.29)	-3.794	<0.001*

Data are presented as M (IQR25-75).

*p<0.05. Bolded text indicates that the value is statistically different.

### 3.3 Comparison of baseline cognitive function between MDD and HC groups

**Figure 2:**
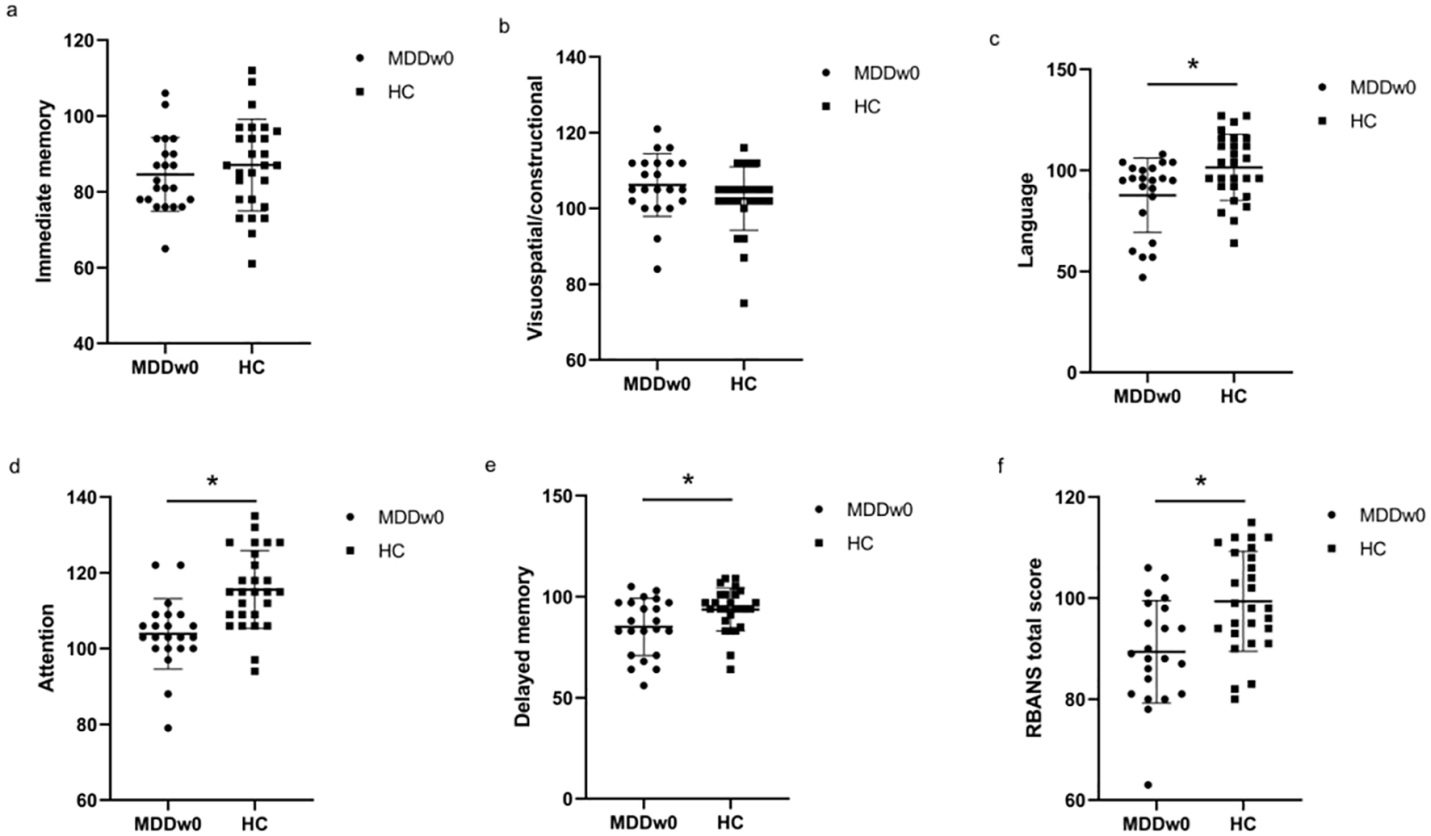
Comparison of baseline cognitive function between MDD and HC groups. *p<0.05. **(A)** Comparison of baseline Immediate memory between MDD and HC groups. **(B)** Comparison of baseline Visuospatial/constructional between MDD and HC groups. **(C)** Comparison of baseline Language between MDD and HC groups. **(D)** Comparison of baseline Attention between MDD and HC groups. **(E)** Comparison of baseline Delayed memory between MDD and HC groups. **(F)** Comparison of baseline RBANS total score between MDD and HC groups.

Immediate memory score and visuospatial/constructional ability score showed no significant differences between the MDD and HC groups(*p*>0.05). However, when comparing the language score, attention score, delayed memory score, and RBANS total scale score between the two groups, significant differences were found (*p*<0.05). The MDD group scored lower than the HC group in these areas (**Table 3**, **Figure 2**).

**Table 3:** Comparison of baseline cognitive function between MDD group and HC group.

RBANS	MDD (*n*=22)	HC (*n*=27)	*t*	*P*
Immediate memory,mean (SEM)	84.59 (9.73)	87.07 (12.12)	0.778	0.441
Visuospatial/constructional,mean (SEM)	106.18 (8.30)	102.63 (8.38)	-1.482	0.145
Language,mean (SEM)	87.68 (18.33)	101.48 (16.40)	2.779	0.008*
Attention,mean (SEM)	103.91 (9.26)	115.59 (10.28)	4.135	<0.001*
Delayed memory,mean (SEM)	85.09 (14.10)	93.70 (10.60)	2.440	0.019*
RBANS total score,mean (SEM)	89.36 (10.17)	100.00 (9.53)	3.737	0.001*

Data are presented as mean (SD). RBANS, The Repeatable Battery for the Assessment of Neuropsychological Status.

*p<0.05. Bolded text indicates that the value is statistically different.

### 3.4 Comparison of inflammatory cytokines and BDNF between post-treatment MDD and HC groups

**Figure 3:**
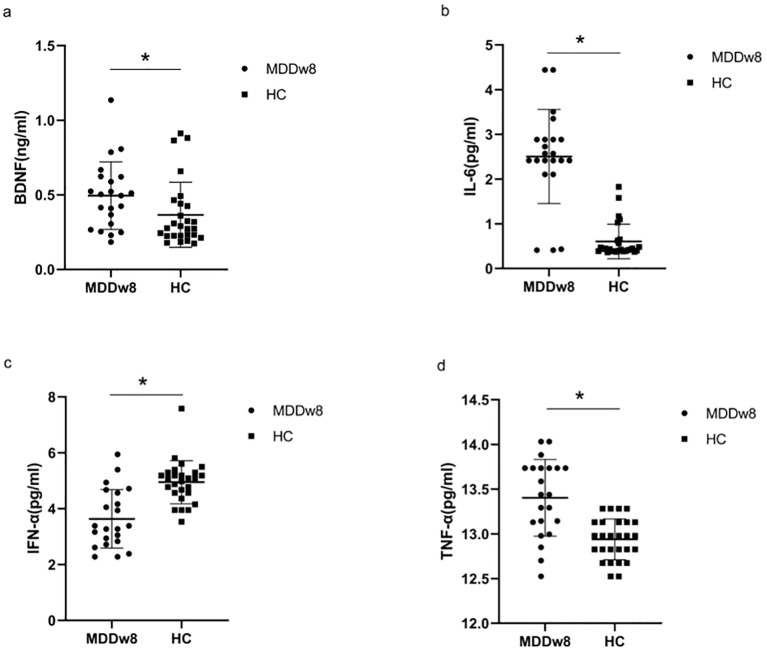
Comparison of inflammatory cytokines and BDNF between post-treatment MDD and HC groups.*p<0.05. **(A)** Comparison of BDNF between post-treatment MDD and HC groups. **(B)** Comparison of IL-6 between post-treatment MDD and HC groups. **(C)** Comparison of IFN-α between post-treatment MDD and HC groups. **(D)** Comparison of TFN-α between post-treatment MDD and HC groups.

There was a statistically significant difference between the post-treatment MDD group and the HC group regarding BDNF, IL-6, TNF-α, and IFN-α levels (*p*<0.05). Specifically, the post-treatment BDNF levels in the MDD group were higher than those in the HC group (*p*<0.05). Additionally, after treatment, IL-6 and TNF-α levels in the MDD group remained exceeded those in the HC group(*p*<0.05).Conversely, IFN-α levels in the post-treatment MDD group were lower than those in the HC group, with the difference being statistically significant (*p*<0.05) (**Table 4**, **Figure 3**).

**Table 4:** Comparison of inflammatory cytokines and BDNF at post-treatment MDD, baseline MDD and HC group.

Index	MDDw0 (n=22) Median(IQR27-75)	MDDw8 (n=22) Median(IQR27-75)	HC (n=27) Median(IQR27-75)	*P_1_*	*P_2_*	*P_3_*
BDNF(ng/mL)	0.31 (0.26, 0.57)	0.50 (0.30, 0.62)	0.28 (0.23, 0.44)	0.338	0.205	0.016*
IL-6(pg/mL)	2.18 (0.41, 2.88)	2.49 (2.34, 2.88)	0.43 (0.39, 0.62)	0.251	0.008*	<0.001*
TNF-α(pg/mL)	13.29 (12.98, 13.59)	13.44 (13.10, 13.74)	12.98 (12.83, 13.13)	0.351	0.001*	<0.001*
IFN-α(pg/mL)	4.05 (3.13, 4.68)	3.33 (2.80, 4.59)	5.08 (4.57, 5.29)	0.112	<0.001*	<0.001*

*p_1_*, Statistical values for baseline MDD comparison after treatment MDD; *p_2_*, Statistical values for baseline MDD compared with HC; *p_3_*, Statistical values for after treatment MDD compared with HC. Bolded text indicates that the value is statistically different. *p<0.05.

### 3.5 Comparison of inflammatory cytokines and BDNF at post-treatment MDD and baseline MDD

**Figure 4:**
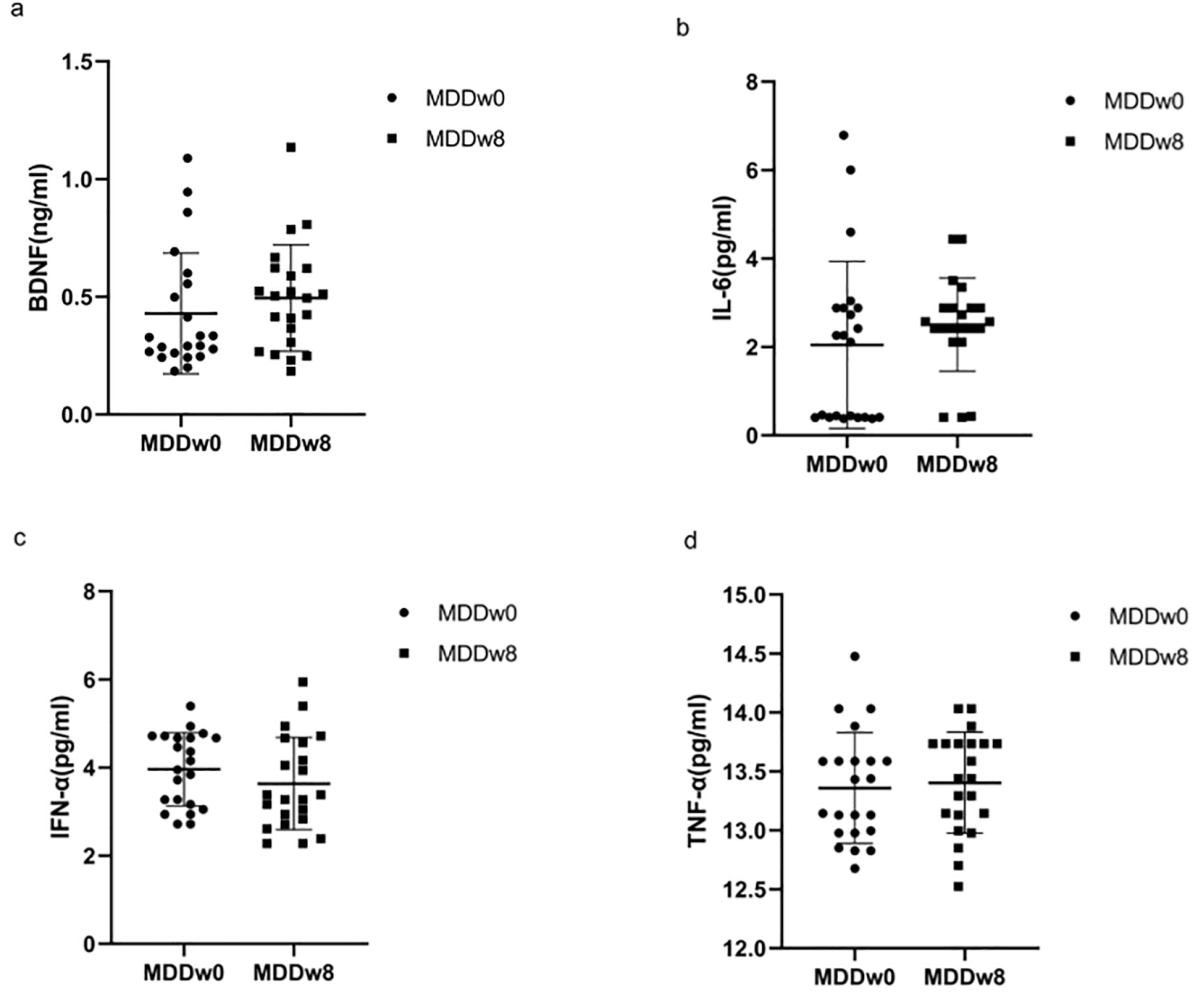
Comparison of inflammatory cytokines and BDNF at baseline MDD, post-treatment MDD. **(A)** Comparison of BDNF at baseline MDD, post-treatment MDD. **(B)** Comparison of IL-6 at baseline MDD, post-treatment MDD. **(C)** Comparison of IFN-α at baseline MDD, post-treatment MDD. **(D)** Comparison of TFN-α at baseline MDD, post-treatment MDD.

There was no statistically significant difference in the levels of BDNF, IL-6, TNF-α, and IFN-α between the MDD group at baseline and after treatment (*p*>0.05) (**Table 4**, **Figure 4**).

### 3.6 Comparison of HAMD-17 and cognitive function between post-treatment MDD and HC groups

**Figure 5:**
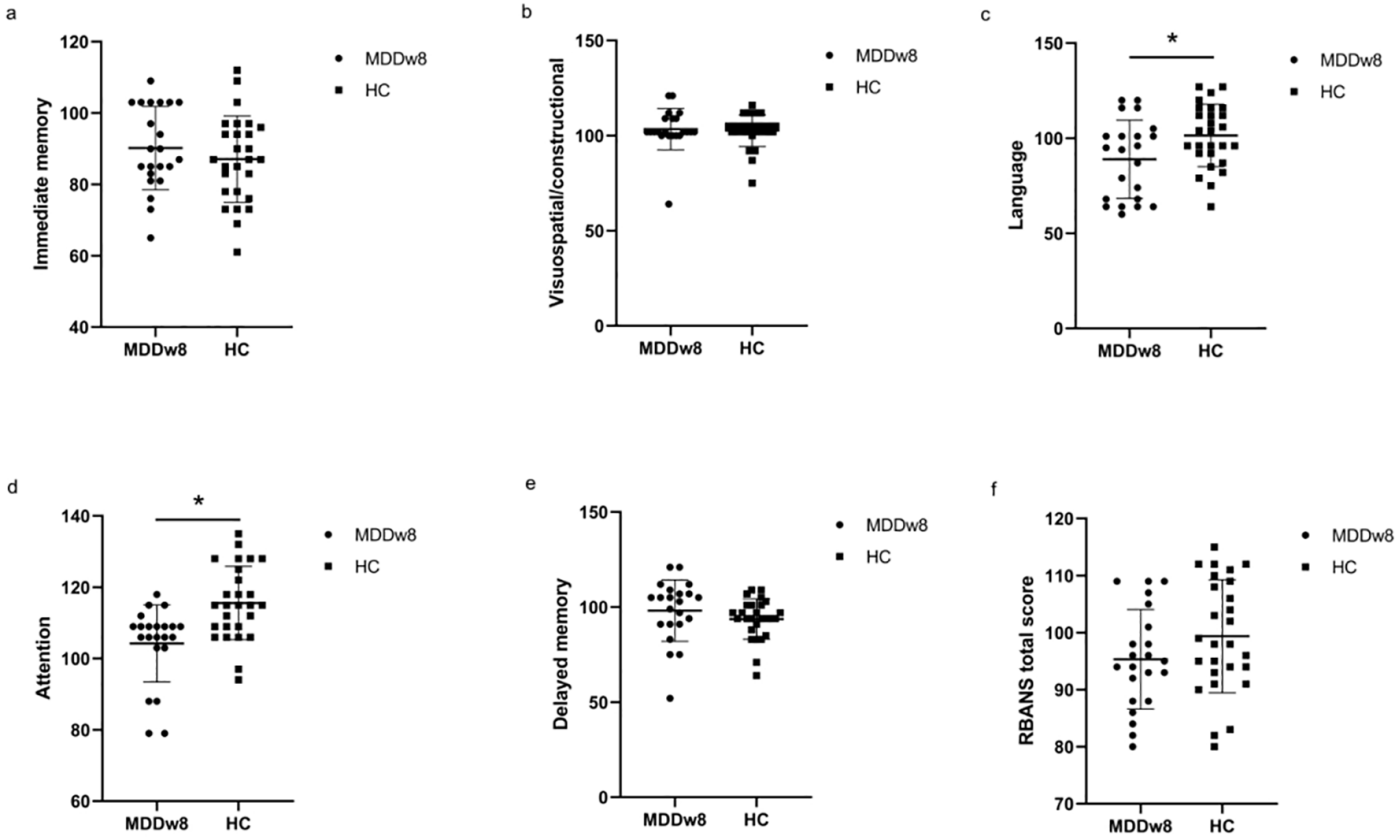
Comparison of cognitive function between post-treatment MDD and HC groups. *p<0.05. **(A)** Comparison of Immediate memory between post-treatment MDD and HC groups. **(B)** Comparison of Visuospatial/constructional between post-treatment MDD and HC groups. **(C)** Comparison of Language between post-treatment MDD and HC groups. **(D)** Comparison of Attention between post-treatment MDD and HC groups. **(E)** Comparison of Delayed memory between post-treatment MDD and HC groups. **(F)** Comparison of RBANS total score between post-treatment MDD and HC groups.

The post-treatment anxiety/somatic factor, cognitive impairment factor, clogging factor, Sleep disorders and total HAMD-17 scores of the MDD group were higher than those of the HC group, showing a statistically significant difference (*p*<0.05).Conversely, the post-treatment language score and attention score of the MDD group were inferior to those of the HC group, with the difference being statistically significant (*p*<0.05) (**Table 5**, **Figure 5**).

**Table 5:** Comparison of cognitive function at post-treatment MDD, baseline MDD and HC group.

	MDDw0 (*n*=22)	MDDw8 (*n*=22)	HC (*n*=27)	*P_1_*	*P_2_*	*P_3_*
HAMD-17						
Anxiety/Somatic Median(IQR27-75)	7 (5, 8)	3 (1, 3.25)	0 (0, 1)	<0.001*	<0.001*	<0.001*
WeightMedian(IQR27-75)	0 (0, 1)	0 (0, 0)	0 (0, 0)	0.114	0.053	0.863
Cognitive impairmentMedian(IQR27-75)	3 (3, 4)	1 (0, 2.25)	0 (0, 0)	<0.001*	<0.001*	<0.001*
CloggingMedian(IQR27-75)	6 (5, 7)	2 (1, 3.25)	0 (0, 1)	<0.001*	<0.001*	<0.001*
Sleep disordersMedian(IQR27-75)	3.5 (2.75, 5)	2 (0, 2)	0 (0, 1)	<0.001*	<0.001*	0.012*
HAMD Total scoreMedian(IQR27-75)	19.5 (17, 22)	9 (3.75, 11)	1 (0, 3)	<0.001*	<0.001*	<0.001*
RBANS						
Immediate memory,mean(SEM)	84.59(9.73)	90.18(11.69)	87.07(12.12)	0.073	0.441	0.408
Visuospatial/constructional,mean(SEM)	106.18(8.30)	103.41(10.89)	102.63(8.38)	0.218	0.145	0.599
Language,mean(SEM)	87.68(18.33)	89.00(20.56)	101.48(16.40)	0.862	0.008*	0.043*
Attention,mean(SEM)	103.91(9.26)	104.23(10.82)	115.59(10.28)	0.808	<0.001*	0.001*
Delayed memory,mean(SEM)	85.09(14.10)	98.18(16.21)	93.70(10.60)	0.007*	0.019*	0.106
RBANS total score,mean(SEM)	89.36(10.17)	95.32(8.73)	100.00(9.53)	0.005*	0.001*	0.142

*p_1_*, Statistical values for baseline MDD comparison after treatment MDD; *p_2_*, Statistical values for baseline MDD compared with HC; *p_3_*, Statistical values for after treatment MDD compared with HC.

*p<0.05. Bolded text indicates that the value is statistically different.

### 3.7 Comparison of HAMD-17 and cognitive function at post-treatment MDD, baseline MDD

**Figure 6:**
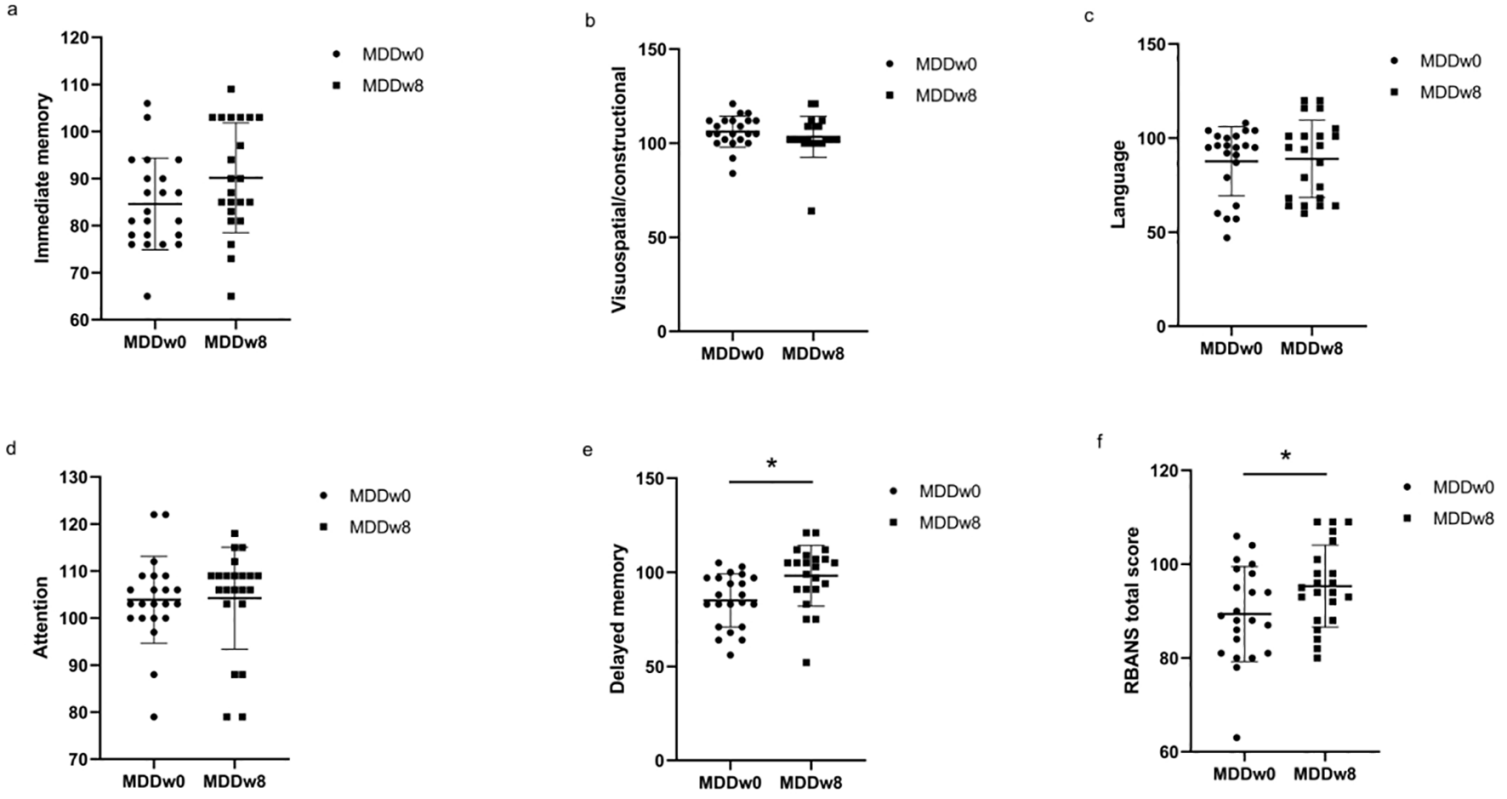
Comparison of cognitive function at baseline MDD and post-treatment MDD. *p<0.05. **(A)** Comparison of Immediate memory at baseline MDD and post-treatment MDD. **(B)** Comparison of Visuospatial/constructional at baseline MDD and post-treatment MDD. **(C)** Comparison of Language at baseline MDD and post-treatment MDD. **(D)** Comparison of Attention at baseline MDD and post-treatment MDD. **(E)** Comparison of Delayed memory at baseline MDD and post-treatment MDD. **(F)** Comparison of RBANS total score at baseline MDD and post-treatment MDD.

The post-treatment anxiety/somatic factor, cognitive impairment factor, clogging factor, sleep disorders factor, and total HAMD-17 scores for the MDD group were lower than their baseline scores, with a significant difference (*p*<0.05). In detail, the post-treatment delayed memory score and RBANS total scale score of the MDD group exceeded those of its baseline, and this difference was statistically significant (*p*<0.05) (**Table 5**, **Figure 6**).

### 3.8 Correlation of inflammatory cytokines, BDNF and cognitive function

At baseline, BDNF was negatively correlated with visuospatial/constructional ability score (r_s_=-0.437, *p*<0.05) and positively correlated with language score (r_s_=0.607, *p*<0.05) in MDD patients; TNF-α was positively correlated with attention score (r_s_=0.518, *p*<0.05).

**Figure 7:**
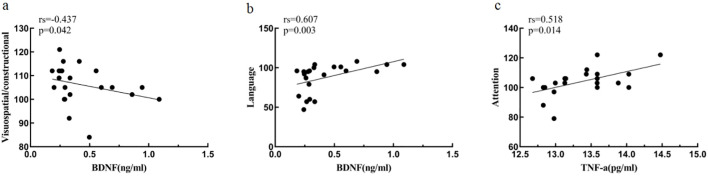
Association of inflammatory cytokines, BDNF and cognitive function at baseline MDD. **(A)** Correlation between Visuospatial/constructional and BDNF at baseline MDD. **(B)** Correlation between Language and BDNF at baseline MDD. **(C)** Correlation between Attention and TNF-a at baseline MDD.

After treatment, no correlation between inflammatory cytokines, BDNF and cognitive function was found for the time being (**Table 6**, **Figure 7**).

**Table 6:** Association of inflammatory cytokines, BDNF and cognitive function.

	BDNF_0_ (ng/mL)	IL-6_0_ (pg/mL)	TNF-α_0_ (pg/mL)	IFN-α_0_ (pg/mL)	BDNF_8_ (ng/mL)	IL-6_8_ (pg/mL)	TNF-α_8_ (pg/mL)	IFN-α_8_ (pg/mL)
Immediate memory_0_	0.071	0.232	0.351	-0.008	–	–	–	–
Visuospatial/constructional_0_	-0.437^*^	0.143	-0.297	0.101	–	–	–	–
Language_0_	0.607^**^	0.019	0.320	-0.185	–	–	–	–
Attention_0_	0.148	0.198	0.518^*^	-0.316	–	–	–	–
Delayed memory_0_	0.117	0.343	0.299	-0.213	–	–	–	–
RBANS total score_0_	0.256	0.214	0.394	-0.202	–	–	–	–
Immediate memory_8_	–	–	–	–	0.068	0.031	0.047	-0.156
Visuospatial/constructional_8_	–	–	–	–	-0.140	-0.250	-0.259	0.325
Language_8_	–	–	–	–	-0.106	-0.024	-0.102	-0.162
Attention_8_	–	–	–	–	0.027	0.222	0.202	0.224
Delayed memory_8_	–	–	–	–	-0.070	0.238	0.275	-0.253
RBANS total score_8_	–	–	–	–	-0.091	0.081	0.041	-0.046

- Indicates no data here *p<0.05, **p<0.01. Bolded text indicates that the value is statistically different.

### 3.9 Multiple linear regression analysis of inflammatory cytokines, BDNF and cognitive function

At baseline, controlling for confounders such as gender, age, years of education and marital status, the multiple linear regression equation with BDNF and inflammatory cytokines as independent variables and with attention as the dependent variable: *F* (4, 17) = 3.583, *p*<0.05, adjusted R^2^ = 0.330 (**Table 7**); the rest of the regression models with immediate memory, visuospatial/constructional, language and delayed memory as the dependent variable respectively were not statistically significant (*p* > 0.05).

**Table 7:** Multiple linear regression analysis of inflammatory cytokines, BDNF and cognitive function.

Dependent variable	Independent variable	*B* (95%*CI*)	*SE*	*β*	*t*	*P*
Attention_0_	TNF-α	11.652 (2.755, 20.548)	4.217	0.590	2.763	0.013*
	BDNF	-13.287 (-1522.760,98.841)	7.465	-0.368	-1.780	0.093
	IL-6	-0.690 (-3.407,2.026)	1.288	-0.141	-0.536	0.599
	IFN-α	-4.464 (-10.990,2.063)	3.093	-0.399	-1.443	0.167

*p<0.05. Bolded text indicates that the value is statistically different.

At post-treatment, controlling for confounders such as gender, age, years of education and marital status, the multiple linear regression equation with BDNF and inflammatory cytokines as independent variables and with immediate memory, visuospatial/constructional, language, attention, and delayed memory as the dependent variable respectively were not statistically significant (*p* > 0.05).

## 4 Discussion

In this study, we discovered differences in inflammatory cytokines (IL-6,TNF-α,IFN-α) and cognitive function (language, attention, delayed memory, and RBANS total score) between patients with MDD and HC at baseline. The baseline IL-6, TNF-α of the MDD group were higher than those of the HC group. Conversely, the baseline IFN-α of the MDD group were inferior to those of the HC group; the baseline language score, attention score, delayed memory score, and RBANS total score of the MDD group were lower than the HC group. There was a correlation between IFN-α and attention function, and a correlation between BDNF and visuospatial/constructional, language function in MDD at baseline. Additionally, we found a linear regression relationship between TNF-α levels and attentional function in these patients at baseline by multiple linear regression analysis. After treatment, differences remained in inflammatory cytokines (IL-6,TNF-α,IFN-α), BDNF, and attentional function between MDD patients and HC. However, no correlation was observed between inflammatory cytokines, BDNF, and cognitive function post-treatment.

Our study revealed that after 8 weeks of paroxetine hydrochloride tablets-based medication, HAMD-17 scores decreased in patients with MDD but remained distinct from those of healthy individuals. This suggests an alleviation in the severity of depressive symptoms in MDD patients, aligning with findings from previous studies (32, 33). Ciprian et al. (34) showed that the efficacy of Agomelatine, amitriptyline, escitalopram, Mirtazapine, paroxetine, venlafaxine and votioxetine was superior to other antidepressants. A study found that improvements in cognitive function in patients with depressive disorders treated with antidepressants were positively correlated with reduced salivary cortisol levels, suggesting that antidepressants may be able to improve cognitive function by normalizing hypothalamic-pituitary-adrenal axis activity (35, 36).

We found that language scores, attention scores, delayed memory scores, and RBANS total scale scores were lower in MDD patients than in HC at baseline. Structural neuroimaging indicates that the gray matter volume in the dorsal anterior cingulate cortex (dACC) and right dorsolateral prefrontal cortex (DLPFC) is diminished in patients with MDD, leading to reduced executive function (37, 38). The cytokine hypothesis of depression proposes that inflammatory cytokines can damage the CNS through the blood-brain barrier (12), and chronic stress-induced inflammatory cytokines may remodel or harm cerebral vasculature, thereby impacting cognitive function in MDD patients (38–40). Furthermore, functional MRI study finds peripheral inflammatory cytokines associated with altered functional connectivity of reward processing, cognitive control neural circuits in depressed patients (41). We also observed that post-treatment language scores, attention scores in MDD patients were still lower than in healthy subjects, implying that linguistic deficits, attentional deficits in MDD patients might continue even after depressive symptoms have subsided, aligning with earlier findings (42, 43).

At baseline, IL-6 and TNF-α levels were elevated in the MDD group relative to the HC group. Numerous studies have documented notably higher IL-6 and TNF-α levels in the peripheral blood of MDD patients (44, 45). This may be due to the involvement of inflammatory cytokines in the pathogenesis of depression by modulating the metabolism of monoamine neurotransmitters and influencing neuroendocrine functions (46). However, our study revealed that there was no significant difference in the levels of IL-6,TNF-α between the MDD group at baseline and after treatment and post-treatment IL-6 and TNF-α levels in the MDD group remained higher than in the HC group, diverging from prior study outcomes (47, 48). This discrepancy might be due to the fact that patients with MDD did not experience complete remission of symptoms after 8 weeks of treatment, as well as the clinical attributes of the participants (49, 50).

The present study discovered a correlation between the inflammatory cytokines TNF-α and attention at baseline, suggesting that TNF-α could serve as an objective biomarker for evaluating attention function. There are contrasting findings regarding the impact of TNF-α levels on cognitive function. Some research indicates that elevated TNF-α levels harm brain nerves, culminating in cognitive dysfunction (41, 51). Conversely, other studies argue that TNF-α provides a neuroprotective effect and positively correlates with attention (52). The divergent results might stem from TNF signaling through two distinct receptors, TNF-α RI and TNF-α Rα. Specifically, TNF-RI facilitates cellular death and is tied to neurodegenerative disorders, whereas TNF-αRα primarily promotes tissue regeneration and neuroprotection (53, 54). Furthermore, soluble human TNF-α Rα agonists have been shown to shield dopaminergic neuronal cells from oxidative stress in an *in vitro* Alzheimer’s disease model (55).

IFN-α is predominantly generated by leukocytes and possesses antiviral and immunomodulatory properties. Research has indicated that certain hepatitis C patients develop major depression post-IFN-α treatment, potentially due to IFN-α can significantly down-regulate the expression of S100A10 protein in the hippocampus and cingulate gyrus of the brain, and the decrease of S100A10 protein leads to the decrease of the level of 5-hydroxytryptamine receptor 1b/4, which causes the impairment of synaptic 5-hydroxytryptamine signaling in the relevant neuronal nuclei, and ultimately leads to the development of depression in the organism (56). Moreover, post-IFN-α treatment, IFN-α stimulates indoleamine 2,3-dioxygenase, activating the kynurenine pathway. This results in diminished 5-HT synthesis and a notable drop in 5-HT concentration (57, 58), factors intrinsically linked to the onset of major depression. In our research, we noted lower IFN-α levels in the MDD group compared to the HC group at the outset. The reason for this situation may be the presence of immune dysfunction in MDD patients, and low levels of IFN-α have the immunomodulatory function of activating monocytes and/or macrophages to enhance the production of various cytokines (e.g., TNF-α, IL-1, IL-6, etc.) by both (59, 60). In addition, although some IFN-α interventions can lead to depression, it does not mean that depression is always characterized by elevated IFN-α levels, and the reduced IFN-α levels in the MDD patients in our study may just be a manifestation of the immune dysfunction in the MDD patients, and the 8-week treatment still did not change this dysfunction.

In this study, we observed elevated BDNF levels in treated MDD patients. BDNF is a neurotrophic factor essential for neuronal survival, growth, and neuroplasticity, with its levels correlating with neurocognitive function (61). We chose SSRIs as antidepressants for this study. These antidepressants directly bind to TrkB, leading to increased BDNF levels (62). Conversely, transglutaminase catalyzes 5-HT to diminish downstream 5-HT signaling and results in decreased frontal cortical BDNF receptor levels (63). SSRI analogues, however, elevate interneuron 5-HT levels, and the consumption of transglutaminase by 5-HT causes an upsurge in BDNF receptor levels.

## 5 Limitations

To our knowledge, many studies on cognitive function in first-episode major depressive disorder have been cross-sectional. Only a handful have explored the longitudinal relationships between cognitive function and both inflammatory factors and BDNF in first-episode major depressive disorder (64–66). In our research, we delved into the association between cognitive function, inflammatory factors, and BDNF by monitoring changes in cognitive function, inflammatory factors, and BDNF in MDD patients following 8 weeks of pharmacological treatment. Nevertheless, this study is not without its limitations. Firstly, the limited sample size and short follow-up period suggest the need for larger samples as well as longer follow-up periods in subsequent studies. Secondly, given the heterogeneity of major depressive disorder, future research should consider subtyping the condition into forms like melancholic depressive disorder or anxious depressive disorder to develop more tailored treatment plans. Thirdly, this study only scrutinized the relationships between BDNF, IL-6, TNF-α, IFN-α, and the RBANS Scale. Expanding research indicators in the future could help identify factors influencing cognitive enhancement post-treatment. Lastly, it is possible that any learning effects may have influenced on higher RBANS scores after 8 weeks of treatment, which may result in no objective biomarkers associated with post-treatment cognitive improvement.

## 6 Conclusions

By examining correlations between cognitive function and BDNF, IL-6, TNF-α, and IFN-α both at baseline and after 8 weeks in MDD, we determined a link between BDNF and both visual span and verbal function at the outset. We also found an association between TNF-α and attentional function initially, suggesting TNF-α could potentially serve as an objective biomarker to support the assessment of attentional function at baseline. However, we did not identify any objective biomarkers associated with post-treatment cognitive improvement.

## Data Availability

The raw data supporting the conclusions of this article will be made available by the authors, without undue reservation.
